# Identifying Core Regions for Path Integration on Medial Entorhinal Cortex of Hippocampal Formation

**DOI:** 10.3390/brainsci10010028

**Published:** 2020-01-05

**Authors:** Ayako Fukawa, Takahiro Aizawa, Hiroshi Yamakawa, Ikuko Eguchi Yairi

**Affiliations:** 1 Graduate School of Science and Engineering, Sophia University, 7-1 Kioi-cho, Chiyoda-ku, Tokyo 102-8554, Japan; i.e.yairi@sophia.ac.jp; 2 Graduate School of Medicine and Faculty of Medicine, The University of Tokyo, 7-3-1 Hongo, Bunkyo-ku, Tokyo 113-0033, Japan; tak.1829.aiz@gmail.com; 3 The Whole Brain Architecture Initiative, a Specified Nonprofit Organization, Nishikoiwa 2-19-21, Edogawa-ku, Tokyo 133-0057, Japan; ymkw@wba-initiative.org; 4 Dwango Co., Ltd., KABUKIZA TOWER, 4-12-15 Ginza, Chuo-ku, Tokyo 104-0061, Japan

**Keywords:** path integration, hippocampus, medial entorhinal cortex, grid cell, place cell, speed cell, head-direction cell, navigation, cognitive map

## Abstract

Path integration is one of the functions that support the self-localization ability of animals. Path integration outputs position information after an animal’s movement when initial-position and movement information is input. The core region responsible for this function has been identified as the medial entorhinal cortex (MEC), which is part of the hippocampal formation that constitutes the limbic system. However, a more specific core region has not yet been identified. This research aims to clarify the detailed structure at the cell-firing level in the core region responsible for path integration from fragmentarily accumulated experimental and theoretical findings by reviewing 77 papers. This research draws a novel diagram that describes the MEC, the hippocampus, and their surrounding regions by focusing on the MEC’s input/output (I/O) information. The diagram was created by summarizing the results of exhaustively scrutinizing the papers that are relative to the I/O relationship, the connection relationship, and cell position and firing pattern. From additional investigations, we show function information related to path integration, such as I/O information and the relationship between multiple functions. Furthermore, we constructed an algorithmic hypothesis on I/O information and path-integration calculation method from the diagram and the information of functions related to path integration. The algorithmic hypothesis is composed of regions related to path integration, the I/O relations between them, the calculation performed there, and the information representations (cell-firing pattern) in them. Results of examining the hypothesis confirmed that the core region responsible for path integration was either stellate cells in layer II or pyramidal cells in layer III of the MEC.

## 1. Introduction

The hippocampal formation is the brain region that controls the memory of vertebrate animals. This region is deeply involved in spatial memory, such as spatial learning and spatial searching. Actions that require spatial memory for appropriate route selection while obtaining environmental information in a space and reaching destinations are called “navigation”. As a psychological concept closely related to navigation, Tolman proposed the idea of a “cognitive map” [1,2,3]; animals use the map formed with the spatial position of the environment acquired by their exploration. In the field of neuroscience, cells whose firing rate is modulated by a spatial position in the environment were found in the rodent hippocampus and named “place cells” [4,5]. This discovery led to the thought that the hippocampus plays an essential role in the formation of cognitive maps in mammals. As a premise for executing navigation and forming a cognitive map, “self-localization”, which is the ability of animals always to recognize their current position, is indispensable. There are several methods for self-localization. “Path integration” is one of them, and it is a function of animals to calculate their position by integrating their movement information. The core region responsible for path integration is the medial entorhinal cortex (MEC) in the hippocampal formation [6]. MEC has “grid cells” that fire simultaneously with place cell firing when an animal is in a specific place in the environment [7]. Grid cells are involved in realizing path integration; both they and place cells are present in different places in the hippocampal formation, and the number of cells that fire is different [7].

Grid cells have been researched on neuroscientific models and computational models [8]. Both grid cell models that have a relationship without [9,10,11,12,13] and with [14,15,16,17,18,19] path integration have been proposed. There are two famous examples of neuroscientific models: Oscillatory interference models [20,21,22] and continuous attractor network models [6,23,24,25,26,27,28,29]. In response to the experimental discovery of a recurrent structure involving inhibitory interneurons in the connection of grid cells at the MEC [30,31], an improved continuous attractor network model was proposed in recent years [32]. By combining oscillatory interference models and continuous attractor network models, a new model of continuous attractor networks that generate phase precession was also constructed [33,34]. In the field of computational model studies, expressions similar to grid cells appeared in machine learning systems that were trained to perform path integration [14,35]. A vector-based grid model inspired by this machine learning system was also created [36]. Meanwhile, simultaneous localization and mapping (SLAM) is famous as an engineering technique for self-localization. SLAM is a technology in which robots autonomously construct environment maps and realize autonomous behavior through the simultaneous execution of self-localization and map construction. There are two types of SLAM techniques; one emulates the animal hippocampus, and the other does not. An example of the former is RatSLAM, inspired by a navigation system that uses path integration in the hippocampus and MEC of rats [37,38,39,40].

As the involvement of grid cells and hippocampal formation in path integration is clarified, a vast amount of knowledge about spatial memory has been accumulated at the regional, neural circuit, cell-firing-pattern, and neurotransmitter levels with the results of many studies of hippocampal formation with behavioral, physiological, and neuroanatomical experiments. However, these wide-ranging research results, from macro to micro, are highly specialized knowledge that focuses on specific parts at specific levels. As far as we know, there is no research that combines and integrates the knowledge of several parts at several levels with a focus on spatial memory. The objective of this research is to clarify the detailed structure of the MEC at the cell-firing level, which is the core region responsible for path integration, through a literature survey of wide-ranging research results from macro to micro. Specifically, we created a neural circuit diagram that covers all connections between the hippocampus and MEC using knowledge extensively gathered from behavioral, physiological, and neuroanatomical experiments at the regional, neural circuit, and cell-firing-pattern levels. Furthermore, we collected knowledge of various cell-firing patterns in the hippocampal region and added comprehensive information to the created neural circuit diagram about which firing-pattern cells exist in which region. We also collected knowledge about path integration and clarify the relationship of functions related to path integration, such as cognitive map, navigation, and self-localization. The obtained results clarify the relationship between path integration input and output and the calculation method, and enumerate the required conditions for the execution of path integration. Finally, we clarify the detailed structure of the MEC cell-firing level, which is the core region responsible for path integration, to derive current and prospective positions by searching regions from the created neural circuit diagram that satisfy these conditions.

We detail the literature search method in the next section before outlining the review results. First, this paper draws a neural circuit diagram that covers all connections between hippocampus and MEC, including comprehensive information on which firing-pattern cells exist in which region. The neural circuit diagram added with the cell-firing-pattern information is called the schematic diagram of the neural circuit (SDNC). Second, we outline the relationship of functions related to path integration, such as cognitive map, navigation, and self-localization, by gathering knowledge about path integration. The relationship between path integration input and output, and the calculation method is also clarified. Third, this paper identifies how and where path integration is calculated on the basis of the clarified input, calculation, and output of the path integration. This identification process is as follows: (1) hypothesizing the function of path integration, (2) enumerating the conditions required to execute the function, and (3) verifying the success or failure of the hypothesis on the SDNC. Finally, these review results are discussed.

## 2. Methods

PubMed [41] is a bibliographic database that was used to search for articles. When the following keywords were used, “navigation* AND hippocampus* AND medial entorhinal cortex* AND grid cell* AND path integration*”, only 11 articles were found. The oldest of these articles was published in 2012, and six of them were published after 2015. A search for “navigation* AND hippocampus*” found a total of 1571 articles. A search for “hippocampus* AND medial enteric cortex*” found a total of 1254 articles. Older papers found in these searches were published in the late 1970s. A search for “medial entorhinal cortex* AND grid cell*” found a total of 248 articles. Considering that the grid cell was first discovered in 2005, many research articles about grid cells were found. A search for “grid cell* AND path integration*” found 100 articles. A search for “navigation* AND hippocampus* AND grid cell*” found 109 articles. A search for “navigation* AND hippocampus* AND path integration*” found 83 articles. A search for “navigation* AND hippocampus* AND medial entorhinal cortex* AND grid cell*” found 44 articles. A search for “navigation* AND hippocampus* AND grid cell* AND path integration*” found 22 articles. A search for “navigation* AND hippocampus* AND medial entorhinal cortex* AND path integration*” found 15 articles. There are many papers on hippocampus and navigation. However, as mentioned above, only 11 papers were found when simultaneously searching for the following five search words: “hippocampus” and “medial entorhinal cortex”, which are related to spatial memory; “grid cell”, which represents spatial information; “path integration”, which is required for navigation; and “navigation”.

These 11 papers include a review article on a robotics experiment and modeling [42], and articles on self-organizing maps [43,44,45]. These four articles are not in the field of neuroscience and were excluded in this paper. An article on the effects of the inhibition of neurotransmitter receptors on grid cells [46] and an article on the effects of the lack of specific receptors on path integration and grid cells [47] were also excluded in this paper because they were experimental papers focusing on neurotransmitters. Another review article on the similarities and differences in the hippocampus between rodents and primates [48] was important research but was excluded in this paper to focus on rodents. An article on “phaser cells” and their simulation [49] was also excluded because phaser cells were discovered mainly in the medial septum that is related to the hippocampus. As a result, three articles [50,51,52] were left out of 11 papers.

In addition to the three articles that focused in grid cells that are present in MEC related to path integration, it was indispensable to collect the following information: (1) connection between the hippocampus and the surrounding region, (2) cell-firing patterns other than those of grid cells observed in hippocampal formation, (3) path integration input/output, and (4) functions related to path integration such as navigation and self-localization. By individually inputting keywords with the related search words of the four categories above into PubMed, papers including more detailed or the latest findings and review papers were preferentially investigated.

Input keywords and found results that were related to the regions other than the hippocampus, MEC, and the cell-firing pattern observed in the region were the following: “parasubiculum* AND grid cell*” found 18 articles, and “presubiculum* AND grid cell*” found 12 articles. Input keywords and found results that were related to each cell-firing pattern and the region where the pattern is observed were as follows: “head direction cell* AND medial entorhinal cortex*” found 74 articles, “border cell* AND medial entorhinal cortex*” found 60 articles, and “speed cell* AND medial entorhinal cortex*” found 48 articles. Papers related to each function were also individually searched. Input keywords and results are as follows: “navigation* AND path integration* AND hippocampus*” found 87 articles, “cognitive map* AND path integration* AND hippocampus*” found 25 articles, and “phase precession* AND path integration* AND hippocampus*” found six articles. In addition to these PubMed results, books written by a hippocampal researcher were also searched [5,23,53,54].

Among these search results, papers related to simulation models such as oscillatory interference models and continuous attractor network models were excluded. Articles at levels other than the regional, neural circuit, and cell-firing-pattern levels were also excluded. Since path integration is one of the main functions of animal mobility, articles on animals that are stationary or sleeping were excluded. Although research related to spatial memory and hippocampus has been conducted since the 1970s, grid cells and speed cells were discovered in 2005 and 2015, respectively. Therefore, articles published after 2005 were mainly investigated, and articles published after 2015 were actively searched to obtain new knowledge in this paper. Regarding other cell-firing patterns discovered before 2005, old papers were also investigated. Recent progress in experiment techniques has enabled research regions that were difficult to experiment on and research that focuses on individual cells. As a result of searching for articles, many detailed findings were obtained from the latest papers. A total of 77 papers is reviewed in the next section. The selected papers are roughly divided into two types: Papers on connections and cell-firing patterns and papers on each function, including path integration.

## 3. Results

### 3.1. Neural Circuit Diagram and Cell-Firing-Pattern Information

#### 3.1.1. Connection Relationship between Medial Entorhinal Cortex and Surrounding Region

The hippocampal formation is a part of the limbic system and is divided into the hippocampus, subiculum (Sb), presubiculum (PreSb), parasubiculum (ParaSb), and entorhinal cortex (EC). Hippocampus is further divided into the dentate gyrus (DG), CA1, CA2, and CA3. The hippocampal formation transmits information to various regions of the brain, including the neocortex. Most of the information transmission uses EC as a relay region, which is divided into medial EC (MEC) and lateral EC (LEC), with different input/output and processing functions.

The routes that converge on the hippocampus via EC are the ventral visual stream, which processes object-related information, and the dorsal visual stream, which processes action-related spatial information [55]. The ventral visual stream runs via LEC to the hippocampus, and the dorsal visual stream runs via MEC to the hippocampus. LEC and the perirhinal cortex (PER) projecting to LEC are interconnected with regions such as the amygdala and orbitofrontal cortex that represent information about different rewards and punishers [55]. Neurons in the LEC show weak spatial specificity compared to MEC [56], even in cue-rich environments [57]. Neuron activity in the MEC is correlated with spatial recognition variables such as grid cells and head-direction cells [7,58]. Nonspatial information is conveyed from the LEC, and spatial information is conveyed from the MEC to the hippocampus [56]. Moreover, information integration between MEC and LEC is performed at DG and CA3 in the hippocampus [59,60,61].

Although MEC anatomically forms a I to VI layer structure, this paper defines the following six layers as the information processing unit in MEC: MECI, MECII, MECIII, MECVa, MECVb, and MECVI [53,62,63,64]. The reason for excluding layer IV is that there are few neurons in the IV layer of MEC. Information processing is mainly performed in layers II, III, and V of MEC. Layer V is divided into a thin layer (Va) and a thick layer (Vb). MEC has several types of excitatory cells, mainly pyramidal cells and stellate cells [53]. Pyramidal and stellate cells are also present in ParaSb and PreSb [65]. Inputs to MEC are connections from ParaSb to MECII, from the postrhinal cortex (POR) to MECII via MECI, from PreSb and POR to MECIII, and from the retrosplenial cortex (RSC) to MECVb. ParaSb also receives a projection from the RSC, but the projection is relatively weak compared with PreSb. POR sends new (current) information received from the neocortex [66]. The outputs from MEC are the connections from MECII to DG and CA3, and from MECIII to CA1 and Sb. Moreover, Sb projects to ParaSb and PreSb. These connections have a recurrent structure from a global perspective. CA1 and Sb also project to MECIII and MECVb. Furthermore, CA1 projects to the medial septum, nucleus accumbens, and prefrontal cortex (PFC), as well as to Sb and MEC [67,68,69]. Pyramidal cells in ParaSb and MECII receive the projection from the medial septum, have robust theta rhythmicity [70,71], and are involved in theta-wave generation. The main connections of DG, CA3, CA2, CA1, and Sb are described with reference to the figure by Llorens-Martín et al. [72].

#### 3.1.2. Various Cell-Firing Patterns in Hippocampal Formation

In the hippocampus and surrounding regions, cells that express various types of information were discovered, and functional cell types were identified. Most of them are functional cells related to spatial representation.

Neurons related to environmental localization called place cells were discovered in the hippocampus of rats [4,5]. With this discovery as a starting point, details of the neural mechanisms related to spatial navigation have been clarified. Place cells are neurons that are active only when an animal comes to a specific place in the environment, and do not fire in other places. The activity of place cells is not significantly affected by the animal’s facing direction, or the presence or absence of lighting. Different place cells are active in different places, and environmental maps are thought to be memorized as a neural circuit of those place cells (cognitive map) [5]. Place cells are present in the hippocampus, Sb, ParaSb, and PreSb [73,74]. The hippocampus is also an essential region of environmental localization in humans [75], and the presence of place cells was confirmed [76].

Head-direction (HD) cells that only fire when the head is facing a specific direction regardless of the location of a rat were also discovered [58,77]. These neurons receive self-motion information, such as vestibular sensation, and calculate direction information [54]. HD cells are present in MEC, ParaSb, PreSb, and RSC [74].

Boundary vector cells were discovered on the basis of place cell responses to boundary manipulations [78,79,80]. Border cells, which selectively fire near the boundary, were discovered by experimental research afterward [81,82,83]. Boundary vector cells and border cells have slightly different properties. Boundary vector cells fire across the entire length, while border cells fire along only a portion of the environmental borders [3]. Boundary vector cells and border cells can serve similar functions by stabilizing grid cells and contributing to the formation of hippocampal place fields. Error accumulation in path integration causes a drift. It was suggested that the drift is corrected by temporarily resetting the grid cell signal generated continuously through path integration by synaptic input from border cells [84]. Border cells are present in MEC, Sb, ParaSb, and PreSb [74]. 

Grid cells that fire depending on the environmental position of rats, similarly to place cells, were also discovered [7]. Unlike place cells in the hippocampus, grid cells fire in multiple locations and form a tessellated grid. Grid patterns of different cells vary in scale, orientation, and phase [85,86], but maintain consistent geometric relationships with each other across environments or experiment manipulations [87,88,89]. Grid cells were discovered in the MEC, ParaSb, and PreSb [7,74]. Many pure grid cells in layer II of the MEC have no properties other than those of grid cells [7]. Contrary to this, grid cells localize with HD cells and border cells in layers III–VI of MEC, ParaSb, and PreSb, and many of them have the properties of HD cells and grid cells, conjunctively [58,90]. Grid cells were not only observed in rodents [7], but also in bats [91,92] and humans’ dorsal MEC [93,94,95]. The grid size of grid cells is small (30 cm) on the dorsal MEC and large (more than 3 m) on the ventral MEC [96], and it increases from the dorsal side to the ventral side [7,58,97]. Multiple grid cells create coordinate systems of various scales in the MEC. Many models were created on the basis of the idea that place cells are formed by combinations of grid cells of different scales. Some experiments showed that place cells collapse due to the inhibition of grid cell firing. However, it is known that place cells are able to form even in young rodents whose grid cells are not yet mature [98,99]. Grid cells of rodents were observed to fire from four weeks after birth, but place cells fired approximately 2.5 weeks after birth [51]. There are also some experiment results that show that grid cells are not essential for the formation of place cells in familiar environments [100,101,102]. Therefore, it is not merely claimed that “place cells are formed from grid cells” or “grid cells are formed from place cells”. Expression by grid cells is not a universal requirement for forming place cells or cognitive maps.

In 2015, speed cells were discovered in the MEC by Kropff et al. [103]. Speed cells are neurons that show activity depending on the moving speed of animals. Prospective speed cells were also observed in the MEC of rodents [103]. Although speed cells were observed in all layers of the MEC [103], there is still no certainty about the region where speed information is generated.

The MEC has several types of excitatory cells, such as stellate cells and pyramidal cells, which are classified according to the anatomical structure of the cells [53]. Among them, about 40.1% of stellate cells and about 18.4% of pyramidal cells are grid cells [104]. It is not yet determined whether border cells are stellate cells [105]. Because inactivation of MEC stellate cells is known to impair spatial learning, they are required for spatial learning [106].

#### 3.1.3. Schematic Diagram of Neural Circuit

**Figure 1:**
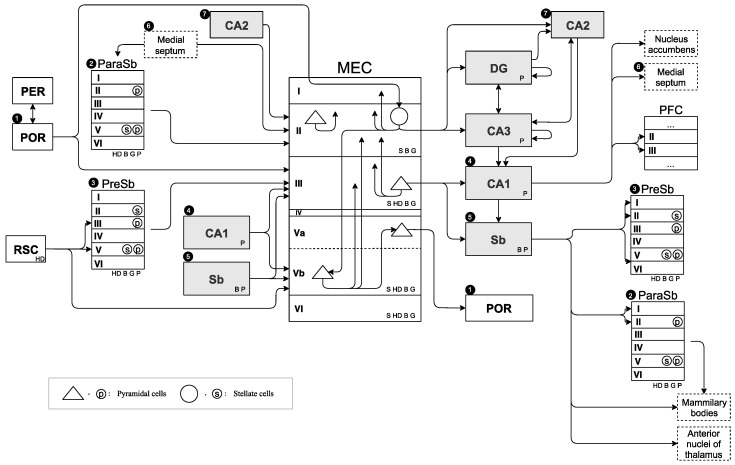
Schematic diagram of neural circuit (SDNC). Single arrow denotes projection in one direction, and double arrow denotes projection in both directions. Arrow starting points of presubiculum (PreSb) and parasubiculum (ParaSb) projected to medial entorhinal cortex (MEC) are slightly away from the square that represents their regions because their output layers are still unclear. However, it is clear that PreSb and ParaSb project to MECII and MECIII, respectively. Dotted squares represent limbic systems other than hippocampal formation, and solid squares represent other regions. Gray squares represent regions without a six-layer structure, dentate gyrus (DG), CA1, CA2, CA3, and subiculum (Sb); white squares represent other regions with layer structures. Some layer structures of the perirhinal cortex (PER), postrhinal cortex (POR), retrosplenial cortex (RSC), and prefrontal cortex (PFC) were omitted. Connection between some other regions was also omitted to precisely describe input/output relationship between MEC and other regions. Numbers 1–7 in black circles denote that same regions were drawn twice on both MEC input and output sides. Stellate cells and pyramidal cells were drawn inside layers of MEC, PreSb, and ParaSb. Cell firing patterns of speed (S), head-direction (HD), border (B), grid (G), and place (P) cells were drawn in regions where they are present. In PreSb and ParaSb, it is not clear which cell-firing pattern exists in which layer, so cell-firing pattern was drawn in the lower right corner of the square of the region.

We created a neural circuit diagram that covered all connections between the hippocampus, MEC, and surrounding regions on the basis of findings in Section 3.1.1. The created neural circuit diagram has comprehensive information on what kind of firing-pattern cells are present in which region in the hippocampal region on the basis of findings from Section 3.1.2. We call this diagram the SDNC, and it is shown in Figure 1. MEC structure was drawn in the center of the figure. The region to MEC input was drawn on the left side of the MEC, and the region output by the MEC was drawn on the right side of the MEC. To emphasize the input/output relationship of MEC, some regions were drawn on both the left and the right side. SDNC contains all the information on the positional relationship of the anatomical region, connection, and type of cell-firing pattern present in them. To create a neural circuit diagram that centers on the MEC, we investigated the existence of connections between all regions related to path integration and the regions adjacent to them. It is crucial to not only specify that there is a connection, but also to specify that there is none.

In Figure 1, the single arrow denotes a projection in one direction, and the double arrow denotes a projection in both directions. The arrow starting points of the PreSb and ParaSb projected to the MEC are slightly away from the square that represents their regions because their output layers are still unclear. However, it is clear that PreSb and ParaSb project to MECII and MECIII, respectively. Dotted squares represent limbic systems other than hippocampal formation, and solid squares represent the other regions. Gray squares represent regions without a six-layer structure, that is, DG, CA1, CA2, CA3, and Sb, and white squares represent other regions with layer structures. For simplicity, some layer structures of PER, POR, RSC, and PFC were omitted in the diagram. The connection between some other regions was also omitted to precisely describe the input/output relationship between MEC and other regions. Numbers 1–7 in black circles denote that the same regions were drawn twice on both the input and output sides of the MEC. Stellate cells and pyramidal cells were drawn inside the layers of MEC, PreSb, and ParaSb. The cell-firing patterns of speed (S), HD, border (B), grid (G), and place (P) cells were drawn in regions where they are present. In PreSb and ParaSb, it is not clear which cell-firing pattern exists in which layer, so the cell-firing pattern was drawn in the lower right corner of the square of the region.

### 3.2. Path Integration and Related Function

#### 3.2.1. Self-Localization

**Figure 2:**
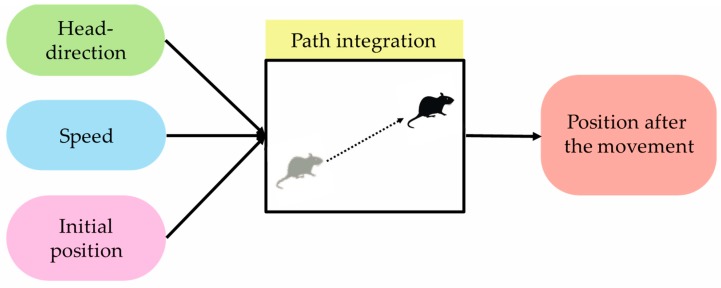
Input and output information that is necessary for path integration. During animal movement in path integration, when initial-position and movement information (head-direction and speed information) is input, position information after movement is output.

Path integration is one of the functions that support self-localization, which is important for animals to move around the environment. In this paper, path integration is defined as a “function that outputs position information after movement when initial-position information and movement information are input” [6]. Movement information here defines head-direction information and speed information. Figure 2 shows path integration and its input/output information. It was confirmed in the field of neuroscience that path-integration calculation is performed using grid cells [50]. Calculation models in the field of computer science indicate that path integration is realized by grid cells in the MEC [8]. However, since grid cells are present in every layer in the MEC, it is not clear which neurons in which layers calculate path integration. 

Error accumulation increases over time because calculation error occurs in path integration in animals [107]. By correcting error accumulation with visual information, more accurate self-localization is performed [106].

#### 3.2.2. Self-Position Correction with Visual Information

**Figure 3:**
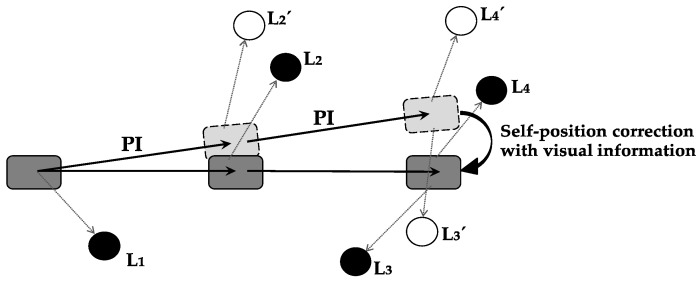
Self-position correction with visual information. PI: Path integration; Ln (black circle): Actual object position; L’n (white circle): Imaginary object position that should be visible from current position obtained by path integration. Dark-gray rectangle represents moving animal and actual position to which the animal is moving. Light-gray rectangle shows animal position obtained by path integration. Calculated position deviates from actual position, since error occurs in the calculation result of animal path integration. Error is corrected using visual-landmark information. Calculation-error size by path integration is difference between white-circle position, indicating imaginary object position, and black circle, indicating actual object position. Correcting this calculation error using visual information and obtaining actual position is self-position correction with visual information in this paper.

Self-position correction with visual information is defined as the function with which the error between current-position-calculation result by path integration and actual position is corrected using visual information. Figure 3 shows a specific example of error correction using visual information. As a result of self-position correction, the accuracy of self-localization is increased, and a precise current position is obtained. 

Self-position correction with visual information must be performed in the region in which path integration results and visual information are integrated. Path-integration information is projected from the MEC to the hippocampus, visual information is projected from the LEC to the hippocampus, and hippocampus integrates both types of information [59,60,61].

#### 3.2.3. Cue-Based Strategies

Depending on the novelty (novel or familiar) and size (large or small) of the environment, self-localization takes multiple strategies with different combinations of path integration and cue-based strategies. Inactivation of the medial septum impairs path integration and suppresses the regular structure in grid cell firing [100,101]. In experiments in a large and novel environment, place cells did not form spatial firing fields when the medial septum was inactivated [108]. It is essential to examine how animals search in large and novel environments [84,109]. In novel environments, animals immediately adopt a “home base” near noticeable landmarks [110,111]. Animals start searching from the home base and then repeatedly and frequently return to the landmark [112]. During exploration, animals intermittently pause and move their heads to gather information about landmarks and other sensory information at that location. Animals can move only a short distance during the initial process of searching but can move a long distance as they continue to search and acquire many landmarks. Animals can reset the path-integration coordinate system by returning to their home base and correcting any small errors before path-integration errors accumulate excessively. By repeating this process, animals can increase the movement range that can be corrected with landmarks. In novel environments, there are no landmarks that help animals to correct the drift caused by path integration because of a lack of memorized visual information. Therefore, for movement, animals need to use path integration and learn landmarks in novel and large environments.

In some cases, when there is enough visual information, animals can move on the basis of known landmarks even if path integration is not functioning. In small [100] and large [108] familiar environments, the firing of place cells in CA3 was destroyed when the medial septum was inactivated in comparison with the case of control animals [113], but place cells in CA1 functioned during medial septum inactivation. In a familiar environment, even if path integration is impaired by medial septum invalidation, it may be possible to maintain the firing field by relying on previously learned associations with landmarks [84]. In an experiment in novel and small environments, the place field of CA1 was normally developed [114]. Furthermore, in small environments, border cells may appear as indistinguishable from grid cells [83]. These facts could explain why border cells reduced the impact on place field formation when path integration was not functioning by suppressing grid cells [84]. The surviving spatial firing in these smaller or familiar environments may be the result of spatial computations based on local landmarks rather than path integration [115,116].

In large and novel environments, path integration is essential. In novel and small or familiar environments, path integration is not crucial for map creation and self-localization. However, animals always continuously perform path integration [84]. Savelli et al. [84] discussed that landmark identification and distance estimation are probably slower perceptual and cognitive processes than numerical integration by path integration. A system that activates path-integration computations only when landmarks become unavailable should not be able to respond to rapid changes in movement. A quick and dirty update from path integration may be better than updates from landmarks in terms of response speed. Path integration is essential even when landmarks are not available in the dark.

#### 3.2.4. Phase Precession

Phase precession is one of the functions required for navigation in animals. It is observed in the hippocampus as the firing order of place cells, which is time-series information of a moving route on the phase of the theta wave [117]. A theta cycle is about 80–250 ms. Past-, current-, and future-position information of rats are compressed and expressed in one theta-wave cycle [118]. Phase precession requires current-, past-, and prospective-position information during one cycle of theta rhythm. The method of calculating this position information is not clarified. Sanders et al. proposed a framework that estimates the current position by path integration and information integration in the hippocampus in the first half of a theta rhythm cycle and predicts the future position (mind travel) in the second half of a cycle of theta rhythm [52]. The relationship between path integration and phase precession is described in their paper. We propose a calculation method that is partly different from the calculation method proposed by Sanders et al. [52].

It was shown that phase precession is observed independently of the hippocampus in spatially modulated grid cells in MECII [119]. Furthermore, phase precession is apparent in nearly all principal cells in MECII, whereas it is sparse in MECIII [119]. Phase precession of grid cells in MECII is not blocked by inactivation of the hippocampus. Therefore, the phase precession could be generated in the grid cell network, and hippocampal phase precession could be inherited from EC [119].

#### 3.2.5. Functional Requirements for Path Integration

Summarizing the investigated results in Section 3.2, we reached the following four conclusions regarding path integration. First, current and prospective positions are calculated in one theta cycle. Second, path integration derives current-position information by calculating the input initial-position information (position information of an earlier theta cycle) and movement information (head-direction and speed information). At this time, position information corrected with visual information of an earlier theta cycle is used as initial-position information that is necessary for path-integration calculation. Third, since path integration accumulates errors [107], self-position correction with visual information modifies these errors and precisely calculates the current position [106]. Therefore, the region where path integration is performed must output the calculation result of path integration to the region where self-position correction with visual information is performed. Since corrected position information is used as initial-position information in the next time, a loop structure, connecting the region where self-position correction with visual information is performed to where path integration is performed, is required to use the correction result as initial-position information. Fourth, prospective position is obtained by path integration when current-position information is input as initial-position information, and prospective-speed information is input as movement information. Unlike current-position calculations that use input from sensory information on moving, future-position calculations require the use of prospective-speed information, which is expected-movement information. Prospective speed cells were experimentally observed in the MEC of rodents [103], although the mechanism for calculating prospective-speed information in the brain has not been revealed.

The four outcomes are not explicitly described in the literature to our best knowledge. In this paper, input/output information of path integration and the relationship between related functions are explicitly demonstrated through the literature survey. These findings may lead to a more detailed structure at the cell-firing level of the MEC, which is the core region responsible for path integration to derive current position and prospective position. Detailed elucidation of this structure promotes a systematic understanding of path integration, and may be useful for brain-inspired artificial intelligence design and new experiment hypotheses in neuroscience. In the next section, this paper clarifies the regions where information input/output and calculations necessary for deriving current position and prospective position are performed. Furthermore, we clarify regions and processes where prospective-speed information necessary for deriving the prospective position is calculated. This paper introduces hypotheses about the function of path integration, enumerates required conditions to execute this function, and verifies the success or failure of the hypothesis on SDNC.

## 4. Ideas on Functional Description of Path Integration

### 4.1. Methodology

**Figure 4:**
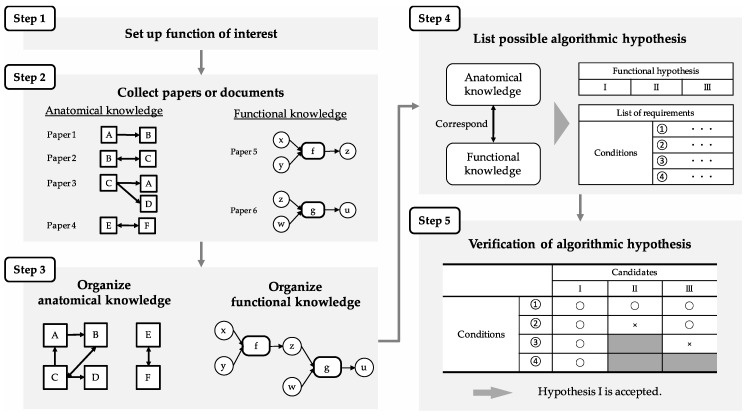
Generalized procedure for finding function-specified region (FSR). Steps 1 to 3 correspond to the review process. In Step 4, the algorithm was constructed, and conditions required for its execution are listed. In Step 5, the algorithm is compared with the SDNC to verify whether region structure, connections, and cell-firing pattern indispensable for execution corresponded to required conditions.

This section proposes a unique method that clarifies which region calculates prospective-speed information and path integration. The detailed descriptions of functional processes in the brain by stepwise refinement, which focuses on region structure and connections and the cell-firing pattern, are hereafter referred to as an algorithm in this paper. Stepwise refinement is used to break down functions into parts and procedures in design engineering and computer science. The algorithm was verified by demonstrating whether either part of the SDNC satisfied the conditions for function execution, such as the structure, connections, and the cell-firing pattern of the region. If no part of the SDNC satisfied all the requirements, the constructed algorithm would be false. Then, the wrong algorithm was undone, and a new algorithm was constructed and verified. This verification was repeated until an algorithm that satisfied the requirements was found. This paper hereafter refers to the part of SDNC that is associated with a correct algorithm as a function-specified region (FSR). In the following subsections, we demonstrate the FSR for calculating prospective-speed information and for calculating path integration on the basis of knowledge on prospective-speed information and path integration that we obtained in Section 3. The function to calculate prospective-speed information is called prospective-speed calculation. Figure 4 shows the procedure of the proposed method, generalized for use in other regions and functions, as well as the MEC and path integration. Steps 1 to 3 correspond to the review process mentioned above. Since path integration is a function of interest in this paper, path integration was investigated and reviewed. In Step 4, the algorithm was constructed, and the conditions required for its execution are listed. In Step 5, the algorithm is compared with the SDNC to verify whether region structure, connections, and cell-firing pattern, indispensable for the execution, corresponded to the required conditions.

The FSR of prospective-speed calculation is first elucidated since it is used for the FSR of path integration. In Step 4, the algorithm of prospective-speed calculation is described, in which the firing pattern of the prospective speed cell is derived from the difference of two simultaneous inputs of positional information at different times. Specifically, it describes the relationship of the region, the calculation performed in the region, the information representation that exists in the region (cell-firing pattern), and the input/output between the regions. Conditions of region structure, connections, and cell-firing patterns are listed as requirements for algorithm execution. Finally, in Step 5, the region that satisfied the conditions was identified by comparing the algorithm with the SDNC. The FSR of path integration was also clarified in the same procedure. In Step 4, algorithms are described for each current- and prospective-position calculation on the basis of the definition of path integration described in Section 3: A “function that outputs position information after movement when initial-position information and movement information (head-direction and speed information) are input”. The prospective-position-calculation algorithm also uses the FSR of the prospective-speed calculation described above. As with prospective-speed calculation, conditions required for path-integration-algorithm execution are listed. Finally, in Step 5, the region that satisfies the condition is identified by comparing the algorithm with the SDNC.

In this paper, some words are defined as follows to be used for explaining the proposed method. Arbitrary time is expressed as *T*, and the place where the animal is at *T* is expressed as *X_T_*. The head-direction information of animals at *T* is expressed as *φ*_T_. The position calculated by path integration before being corrected with visual information, which is the prospective position, is represented by $\hat{X_{T}}$ because it is an estimated position. *V_T_* represents current-speed information, and $\tilde{V_{T}}$ represents prospective-speed information. The time duration of one theta-wave cycle is expressed as Δt. In one theta-wave cycle, the compressed representation of walking path information for several seconds is embedded. Δε is used as the unit of a very short period of time, expressing the location difference between two continuous points within one Δt. Current time is expressed as t. $X_{t}$ is the current-position information that fires at $\sin\frac{\pi }{2}$ of theta cycle (hereafter called “apex”) timing on theta phase precession of the current cycle. $X_{t-1}$ is the past-position information that fires at the apex timing on theta phase precession one cycle earlier (Δt before). $X_{t+1}$ is later-position information that fires at the apex timing on theta phase precession after one cycle (after Δt ). $\hat{X_{t+1}}$ is the prospective information of the next time position that fires at the time after Δε of the apex on the theta phase precession of the current cycle.

### 4.2. Specifying Region for Prospective-Speed Calculation

This section describes the algorithm for prospective-speed calculation and demonstrates the required conditions related to region structure, connections, and cell-firing pattern for algorithm execution. The FSR of prospective-speed calculation was indicated by comparing the algorithm with the SDNC and identifying the region that satisfied the required conditions.

Prospective-speed information enables the calculation of prospective positions. Prospective-speed information is observed as firing pattern of prospective speed cells. Although the firing pattern of prospective speed cells was experimentally observed, it was not clear how prospective-speed information is generated. As a premise, the firing pattern of grid cells in MEC is metric information, and the firing pattern of place cells in the hippocampus is position index without metric information. There is also no evidence that the hippocampus or MEC can keep multiple different instances of speed information at the same time.

**Figure 5:**
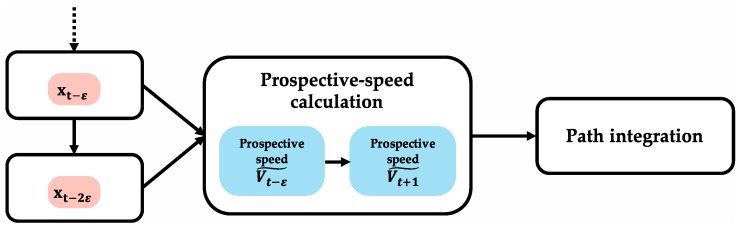
Prospective-speed-calculation algorithm. When two different instances of position information with ε time offset (index information: $X_{t-\varepsilon }$, $X_{t-2\varepsilon }$) are input simultaneously, the two position vectors are obtained by converting position-index information for two adjacent instances into metric information. Prospective-speed information ($\tilde{V_{t-\varepsilon }}$) is obtained by calculating velocity vector from difference between two position vectors. Under this assumption, $\tilde{V_{t-\varepsilon }}$ is expressed as $\tilde{V_{t+1}}$ because $\tilde{V_{t-\varepsilon }}$ represents speed information to calculate next prospective position $\hat{X_{t+1}}$.

Figure 5 shows the algorithm of prospective-speed calculation. In the prospective-speed-calculation algorithm, when two different instances of position information with *ε* time offset (index information: $X_{t-\varepsilon }$, $X_{t-2\varepsilon }$) are input simultaneously, the two position vectors are obtained by converting position-index information for two adjacent times into metric information. Prospective-speed information $\left(\tilde{V_{t-\varepsilon }}\right)$ is obtained by calculating the velocity vector from the difference between the two position vectors. Prospective-speed calculation may be achieved using the newest information of the obtained accurate positions. Therefore, it is assumed that positions at t−ε and t−2ε ($X_{t-\varepsilon }$, $X_{t-2\varepsilon }$) are used in this algorithm. Under this assumption, prospective-speed information $\tilde{V_{t-\varepsilon }}$ is expressed as prospective-speed information $\tilde{V_{t+1}}$ because the prospective-speed information $\tilde{V_{t-\varepsilon }}$ represents speed information to calculate the next prospective position ($\hat{X_{t+1}}$).

Table 1 is a summary of the required conditions for executing the prospective-speed-calculation algorithm. The following three conditions are required for realizing this algorithm. First, there are speed cells that can calculate and output speed information. Second, there are grid cells that can use metric information. Third, there is simultaneous input of two different instances of position-index information.

The region that satisfies the conditions is identified by comparing requirements of prospective-speed calculation listed in Table 1 with the SDNC. Stellate cells and pyramidal cells in layer II of MEC were abbreviated to as MECII-st and MECII-py, respectively. The verification process is as follows. From Condition (1) in Table 1, speed information is necessary to perform prospective-speed calculation, so all regions with speed cells are candidates. Therefore, there are five candidate regions for prospective-speed calculation: MECII-st, MECII-py, MECIII, MECVa, and MECVb. From Condition (2) in Table 1, since grid cells are necessary to convert the index information of place cells into vector information, candidates are narrowed down to the region with grid cells. Candidates remain unchanged: MECII-st, MECII-py, MECIII, MECVa, and MECVb. From Condition (3) in Table 1, the simultaneous input of two different instances of accurate position information, $X_{t-\varepsilon }$ and $X_{t-2\varepsilon }$, from the hippocampus is necessary. Referring to the SDNC (Figure 1), regions that are projected from two different hippocampal regions among the five candidates are only MECIII and MECVb that are projected from CA1 and Sb. Therefore, candidates for prospective-speed calculation are limited to MECIII and MECVb. As the result of narrowing down the prospective-speed-calculation candidate region from the required conditions, MECIII and MECVb remained without rejection. The region responsible for prospective-speed calculation would be MECIII or MECVb. Table 2 summarizes this process.

### 4.3. Specifying Region for Path Integration

This section describes the algorithm for path integration and lists the required conditions related to region structure, connections, and cell-firing pattern for algorithm execution. Furthermore, the FSR of path integration is indicated by comparing the algorithm with the SDNC and identifying the region that satisfies the required conditions. The algorithms for path integration describe each algorithm for the calculation of the current position and prospective position. As a premise, path integration is realized by MEC grid cells. From Section 3, path integration is defined as a function that outputs position information after movement when initial-position information and movement information (head-direction information and speed information) are input.

**Figure 6:**
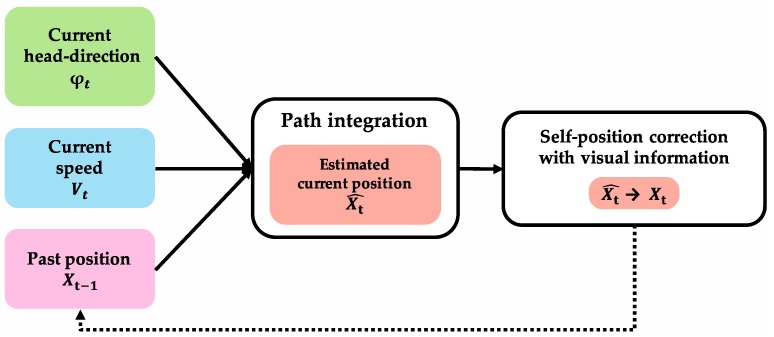
Algorithm of path integration to calculate current position. When initial-position information and movement information are input, position information after movement ($\hat{X_{t}}$) is calculated using grid cells. The region where path integration is performed requires input of head-direction information ($\varphi_{t}$) and speed information ($V_{t}$) as movement information, and past position at one theta cycle before ($X_{t-1}$) as initial-position information. To perform self-position correction with visual information, the region that performs path integration outputs result of path integration ($\hat{X_{t}}$) to region that performs self-position correction with visual information. Corrected position information is assumed to be the initial-position information for the next path integration. This assumption insists that there is a loop structure that inputs correction result ($X_{t}$) as initial-position information from the region where self-position correction with visual information is performed to the region where path integration is performed.

Figure 6 shows the algorithm of path integration that calculates the current position. In the algorithm, when initial-position information and movement information are input, position information after movement ($\hat{X_{t}}$) is calculated using grid cells. The region where path integration is performed requires input of head-direction information ($\varphi_{t}$) and speed information $\left(V_{t}\right)$ as movement information, and past position at one theta cycle before $X_{t-1}$ as initial-position information. To perform self-position correction with visual information, the region that performs path integration outputs the result of path integration $\hat{X_{t}}$ to the region that performs self-position correction with visual information. It is suggested that path-integration information projected from MEC and visual information projected from LEC are integrated at DG and CA3 of the hippocampus [59,60,61]. Therefore, self-position correction with visual information is performed by DG and CA3 of the hippocampus. In addition, corrected position information is assumed to be initial-position information for the next path integration. This assumption insists that there is a loop structure that inputs the correction result ($X_{t}$) as initial-position information from the region where self-position correction with visual information is performed to the region where path integration is performed.

The following four conditions are required for this algorithm. First, grid cells are necessary to execute path integration. Second, there is an output of the path-integration results to the hippocampus (DG, CA3). Third, there is the input of accurate (corrected) position information one time before from the hippocampus. Fourth, there is the input of movement information (head-direction information $\left(\varphi_{t}\right)$ and speed information $\left(V_{t}\right)$).

**Figure 7:**
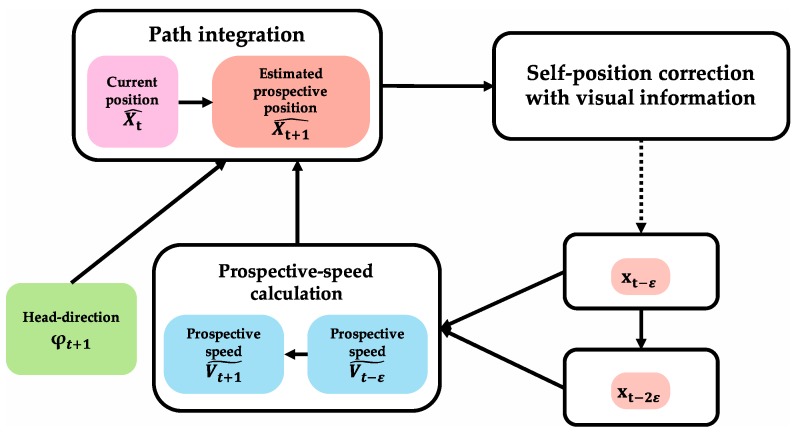
Path-integration algorithm to calculate prospective position. Prospective position is calculated using initial-position information and movement information. In a region where path integration is performed, there is input of movement information (head-direction information ($\varphi_{t+1}$) and speed information ($\tilde{V_{t+1}}$)). Prospective-speed information ($\tilde{V_{t+1}}$) is input from region where prospective-speed calculation is performed. Calculation result of current position, obtained by path integration, is used as necessary initial-position information. There is a loop structure that receives position information ($X_{t-\varepsilon }$, $X_{t-2\varepsilon }$) corrected in the region where self-localization is performed because prospective-speed calculation uses two-position information after error correction. As a result of path integration, prospective position $\hat{X_{t+1}}$ is obtained.

Figure 7 shows the algorithm of path integration that calculates the prospective position. In the algorithm, similar to the calculation of current position, prospective position is calculated using initial-position information and movement information. In the region where path integration is performed, there is an input of movement information (head-direction information ($\varphi_{t+1}$) and speed information ($\tilde{V_{t+1}}$)). Prospective-speed information ($\tilde{V_{t+1}}$) is input from the region where prospective-speed calculation is performed. The algorithm uses the calculation result of the current position obtained by path integration as necessary initial-position information. There is a loop structure that receives position information ($X_{t-\varepsilon }$, $X_{t-2\varepsilon }$) corrected in the region where self-localization is performed because prospective-speed calculation uses two-position information after error correction. As a result of path integration, prospective position $\hat{X_{t+1}}$ is obtained.

The following two conditions are required for this algorithm. First, grid cells are necessary to execute path integration. Second, there is input of movement information (head-direction information ($\varphi_{t+1}$) and speed information ($\tilde{V_{t+1}}$)). Table 3 summarizes these required conditions for calculating current position and prospective position by path integration.

The region that satisfies conditions is identified by comparing requirements of path integration, listed in Table 3 with the SDNC. The verification process is as follows. From Condition (1) in Table 3, all regions with grid cells are candidates because grid cells are required for the execution of path integration. Therefore, there are seven candidate regions for path integration: MECII-st, MECII-py, MECIII, MECVa, MECVb, PreSb, and ParaSb. From Condition (2) in Table 3, path-integration-calculation result must be output to the hippocampus (DG, CA3). Referring to the SDNC (Figure 1), MECII-st has a direct route to the hippocampus (DG, CA3). MECIII, MECVb, PreSb, and ParaSb have an indirect route that outputs to the hippocampus (DG, CA3) via MECII-st. MECII-py and MECVa do not satisfy Condition (2) because they do not have routes to the hippocampus (DG, CA3). Therefore, path-integration candidates are narrowed down to five: MECII-st, MECIII, MECVb, PreSb, and ParaSb. From Condition (3) in Table 3, prospective-speed information needs to be input. From the result of Section 4.2, prospective-speed information is considered to be generated by MECIII or MECVb, so it is necessary to receive projection from at least one of these two regions. Among the five regions selected by Condition (2), MECII-st, with the path projected from both MECIII and MECVb, and MECIII, with the path projected from MECVb, satisfy Condition (3). MECVb does not satisfy the condition. If prospective-speed calculation is performed with MECIII, Condition (3) is not satisfied because there is no projection from MECIII to MECVb. If prospective-speed calculation is performed with MECVb, it is not considered to be involved in prospective-speed calculation and path integration in one region. PreSb and ParaSb do not satisfy Condition (3), because there is no projection from MECIII and MECVb. Therefore, candidates for path integration are narrowed down to MECII-st and MECIII. From Condition (4) in Table 3, the input of corrected position information ($X_{t-1}$) from the hippocampus is required. Referring to the SDNC (Figure 1), MECII-st has an indirect route from Sb via ParaSb. MECIII has direct routes from CA1 and Sb and an indirect route from Sb via PreSb. Therefore, candidates are still MECII-st and MECIII. From Condition (5) in Table 3, the input of HD information is required as movement information. Referring to the SDNC (Figure 1), MECII-st has an indirect route from POR via MECI and an indirect route from POR via MECIII. MECIII has a direct route from POR and an indirect route from RSC via PreSb. Therefore, the candidates are still MECII-st and MECIII. From Condition (6) in Table 3, the input of current-speed information is required as movement information. However, since the generation region of speed information is unclear, the candidate of the region cannot be narrowed down from this condition. As a result of narrowing down the path-integration candidate region from the required conditions, MECII-st and MECIII remained without rejection. Therefore, the region responsible for path integration is assumed to be MECII-st or MECIII. Table 4 summarizes this process.

## 5. Discussion

This paper reviewed extensive knowledge to elucidate the detailed structure at the cell-firing level of MEC that is the core region responsible for path integration. The review results of 77 papers comprehensively scrutinized information on the relationship between hippocampus, MEC, and the surrounding region that is related to path integration, and which firing-pattern cells exist in which region. This paper summarized these findings into a diagram as the SDNC. This summary process is a top-down approach from the viewpoint of drawing an overview of the entire system around the hippocampus and MEC. In addition to region connection and cell-firing pattern, the relationship between input/output information of path integration and related functions was explicitly described by collecting knowledge about path integration. This description is a bottom-up approach from the viewpoint of detailed specification of path integration as individual base elements of animal mobility. Partial neural circuit diagrams of the hippocampus and MEC have been created, but there has never been a diagram that covers the whole hippocampus and MEC. Compared with the diagram of previous research that associated cell-firing-pattern information with regions [74], the created SDNC in this study described regions around the hippocampus and MEC in more detail. There has been no explicit information about input/output information and the relationship of the path-integration function, as far as we know. The SDNC separately describes MEC’s input/output information and draws some regions on both the input and output sides. Therefore, the input/output relationship is easier to understand than a neural circuit diagram drawn in a closed circuit with a loop. The SDNC, with information on the cell-firing pattern of the region, also makes it easy to simultaneously consider where the cell-firing pattern is generated and converted. This study proposed a novel method that combines the results of top-down and bottom-up approaches to identify a region responsible for a function. The proposed method was generalized for use in various brain functions and regions, and then identified regions responsible for prospective-speed calculation and path integration. Finally, prospective-speed-calculation and path-integration algorithmic hypotheses about necessary region, input/output information, and calculation method were created and verified, respectively. As a result, the core region responsible for prospective-speed calculation was narrowed down to MECIII and MECVb, and the core region responsible for path integration was narrowed down to MECII-st and MECIII. Even if the structure of the neural circuit and the location of the cell-firing pattern were revealed by a top-down approach, it would be difficult to clarify how brain functions are carried out. Even if the relationship between functions of interest and related functions were clarified by a bottom-up approach, it would be difficult to identify which regions and cell-firing patterns perform brain functions. By combining these two approaches, this paper clarified the detailed structure at the cell-firing level of MEC, which is the core region responsible for path integration. These results are novel and meaningful contributions.

When integrating comprehensive review findings, a certain amount of knowledge with the same levels of neuroanatomical size (LNS) is required because knowledge of different LNS is not able to be integrated. The amount of knowledge in a certain LNS is sometimes limited because there is little information about regions with experimental difficulty and the current findings. The limitation of this study is that information can only be integrated for LNS with sufficient knowledge. The LNS in this paper consists of the regional, neural circuit, cell-firing-pattern, and neurotransmitter levels. We investigated the first three as they were likely to be fully informed. Relative knowledge imbalance in LNS is difficult to predict before the survey, but it becomes clear in the process of information integration. Literature surveys must fully consider these issues about LNS and the completeness of knowledge. This paper conducted comprehensive information integration with due consideration to these issues as much as possible.

In this paper, knowledge up to the connection level of each layer was sufficiently obtained, but information on the connection level between cells in each layer was not. When creating the SDNC that covers all connections between hippocampus and MEC, we used a strategy in which MEC information processing units were divided into layers as a unit where a certain amount of knowledge was accumulated and in which input/output connection of each layer was discussed. As a result of this paper, the more specific core regions responsible for path integration were narrowed down to MECII-st and MECIII from among many candidates, but could not be specified to one. However, MECII-st could be deeply involved in path integration. Focusing on the internal structure and neurons of the MEC layers, experiment results showed that grid cell calculation is carried on the local circuit composed of pyramidal cells, stellate cells, interneurons of MECII, and pyramidal cells of MECIII [120]. Another result also showed that MEC stellate cells are necessary for spatial learning because inactivation of stellate cells of MEC impairs spatial learning [106]. When considering the internal structure of layers and neurons, MECII-st may be a more specific core region responsible for path integration. However, this is conjecture from the findings of a specific experimental study and only highlights the possibility that MECII-st is a strong candidate. Since there is not an enough knowledge about the internal structure of layers and neurons, it is still difficult to draw conclusions by applying the proposed method in this study.

When further research discovers new knowledge about the internal structure of MEC, cell-firing pattern, and function related to path integration, it will be investigated and updated to add diagrams and refine details. This update enables us to determine more detailed core regions that are responsible for path integration. Knowledge of new experimental research must always be followed to update information. As future development of this research, in order to confirm whether path integration can actually be executed by two candidates for SFRs, we aim to construct an MEC computational model with high neuroscience validity and examine the model by computer simulation.

This approach clarified what kind of knowledge is insufficient. In other words, this approach elucidated what kind of knowledge can be used to determine a more detailed core region responsible for path integration. In this study, more detailed core-region candidates responsible for path integration were narrowed down to MECII-st and MECIII. In order to determine the region from two candidates, the following two neuroscientific experiment hypotheses are provided as examples.

- Whether position information (place cells and grid cells) of ParaSb has information of the past position ($X_{t-1}$).

If past-position information is projected to MECII via ParaSb, representation of position information of ParaSb should have information on past position ($X_{t-1}$). If ParaSb has such information, the possibility of MECII-st candidate is increased. Otherwise, the input of past-position information to the region responsible for path integration is limited to paths that do not go through ParaSb.

- Whether pyramidal cells and interneurons in MECII adjust phase-precession timing in cooperation.

The region responsible for path integration needs to receive different past, current, and future inputs depending on phase-precession timing. It is necessary to confirm how neurons of MECII receiving projection from the medial septum execute to switch processing within one cycle of phase precession. If experiment findings show that pyramidal cells and interneurons in MECII somehow coordinate the phase-precession timing, the possibility of the MECII-st candidate is increased.

The two proposed experiment hypotheses are only examples devised by the authors, and verification methods by other experiments could be considered. The approach of this research is limited by the amount of knowledge obtained from experimental research, but it may lead to an increase in knowledge for subsequent studies by proposing new hypotheses.

The MEC is not limited to self-localization and path integration. Focusing on functions other than path integration, it is possible to hypothesize and verify necessary input/output information and the calculation method for the region responsible for the function of interest by reusing the created SDNC in this paper and using the proposed method. Focusing on functions related to regions other than the MEC and the surrounding regions, the proposed method can be applied in the same way by creating a new SDNC through literature review.

Since knowledge in the field of neuroscience is rapidly increasing, it is possible to clarify specific regions responsible for functions in brain regions other than MEC by using the same approach as this study. The contribution of this research is that this method was proposed as an approach for neurological understanding. Results clarified by the proposed method provide hypotheses to be verified in future research and investigations and can lead to an understanding at the system level of mental disorders and for reference to brain-inspired artificial intelligence. Our achievements could contribute to the development of neuroscience in ways such as those described above.

## 6. Conclusions

This paper reviewed various articles on regional, neural circuit, and cell-firing-pattern levels related to path integration. The neural circuit diagram about the MEC, the hippocampus, and their surrounding regions, named SDNC, was drawn by summarizing the results of exhaustively scrutinizing the papers that are relative to the input/output relationship, the connection relationship, and cell position and firing pattern. The information of functions related to path integration, such as input/output information and the relationship between multiple functions, was also revealed by integrating the knowledge of functions related to path integration. Based on these results, we constructed algorithmic hypotheses on input/output information and calculation method that are required to execute prospective-speed calculation and path integration for self-localization, and enumerated the required conditions for executing functions. The algorithmic hypotheses were verified by comparing the required conditions for executing function with SDNC and concluded that the core region responsible for prospective-speed calculation was MECIII or MECVb, and the core region responsible for path integration was MECII-st or MECIII. However, path-integration candidates could not be specified to one. Although MECII-st may be a more specific core region responsible for path integration, there is not an enough knowledge to determine the region from two candidates. When further research discovers new knowledge about the internal structure of layers and neurons in MEC, it will be investigated to update the information and identify the core regions responsible for functions. Core regions responsible for the other functions than path integration will also be expected to be identified by using the proposed method.

## Figures and Tables

**Table 1:** Required conditions for executing prospective-speed-calculation algorithm.

Condition Type	Condition	Explanation
Presence condition	(1) Speed cells exist.	There are speed cells that can calculate and output speed information.
Presence condition	(2) Grid cells exist.	Position-index information (place cells) is converted into vector information using metric information (grid cells).
Input condition	(3) Two simultaneous inputs from hippocampus.	There is the simultaneous input of two different instances of position information with ε time offset (index information: $X_{t-\varepsilon }$, $X_{t-2\varepsilon }$).

**Table 2:** Process of identifying regions that satisfy required conditions of prospective-speed-calculation algorithm.

Required Condition	Candidate Regions Responsible for Prospective-Speed Calculation				
	MECII-st	MECII-py	MECIII	MECVa	MECVb
(1) Speed cells exist.	YES	YES	YES	YES	YES
(2) Grid cells exist.	YES	YES	YES	YES	YES
(3) There are two simultaneous inputs.	NO	NO	YES	NO	YES
Possible connection			←CA1 and ←Sb		←CA1 and ←Sb

The table describes required conditions and prospective-speed-calculation candidate regions. If a candidate region satisfies the required condition, the region remains as a candidate and shows “YES”. When condition type is input condition, possible connection and region that connect to a candidate region are indicated under the condition. Left arrow (←) indicates input to candidate region. If a candidate region does not satisfy the required condition, the candidate is rejected and shows “NO”. A region once rejected is not evaluated under the following conditions. MECII-st denotes stellate cells in layer II of MEC, and MECII-py denotes pyramidal cells in layer II of MEC.

**Table 3:** Required conditions for executing path-integration algorithm.

Condition Type	Condition	Explanation
Presence condition	(1) Grid cells exist.	Grid cells required for path-integration calculations.
Output condition	(2) Output the calculation result of path integration to the hippocampus (DG, CA3).	Since error correction of path-integration-calculation result ($\hat{X_{t}}$) is performed by the hippocampus (DG, CA3), output of path-integration-calculation result to the hippocampus is required.
Input condition	(3) There is input from region of prospective-speed calculation.	Prospective-position calculation requires prospective-speed information input as movement information.
Input condition	(4) There is input of accurate initial-position information ($X_{t-1}$) at previous time (t−1).	To obtain corrected location information ($X_{t-1}$), the region where path integration is performed needs to receive input from hippocampus that performs self-localization.
Input condition	(5) Head-direction (HD) information input.	HD information input is required as current-movement information.
Input condition	(6) Speed information input.	Current-speed information input is required as movement information. (Generation region of speed information is unclear but was observed in all layers of MEC).

**Table 4:** Process of identifying regions that satisfy required conditions of path-integration algorithm.

Required Condition	Candidate Regions Responsible for Path Integration						
	MECII-st	MECII-py	MECIII	MECVa	MECVb	PreSb	ParaSb
(1) Grid cells exist.	YES	YES	YES	YES	YES	YES	YES
(2) Output to hippocampus.	YES	NO	YES	NO	YES	YES	YES
Possible connection	→DG, CA3		→MECII-st →DG, CA3		→MECII-st →DG, CA3	→MECIII →MECII-st →DG, CA3	→MECII-st →DG, CA3
(3) Prospective-speed information input.	YES		YES		NO	NO	NO
Possible connection	←MECIII or ←MECVb		←MECVb				
(4) Accurate initial-position information $\left(X_{t-1}\right)$ input.	YES		YES				
Possible connection	←ParaSb ←Sb		←CA1 or ←Sb or ←PreSb←Sb				
(5) HD information input.	YES		YES				
Possible connection	←MECI ←POR or ←MECIII ←POR		←POR or ←PreSb ←RSC				
(6) Speed information input.	−		−				
Possible connection	NA		NA				

The table describes required conditions and path-integration candidate regions. If a candidate region satisfies the required condition, the region remains as candidate and shows “YES”. When condition type is an input/output condition, possible connection and region that connect to the candidate region are indicated under the condition. Left arrow (←) indicates input to candidate region, and right arrow (→) indicates output from candidate region. If a candidate region does not satisfy the required condition, the candidate is rejected and shows “NO”. A region once rejected is not evaluated under the following conditions.
